# Lack of virological and serological evidence for continued circulation of highly pathogenic avian influenza H5N8 virus in wild birds in the Netherlands, 14 November 2014 to 31 January 2016

**DOI:** 10.2807/1560-7917.ES.2016.21.38.30349

**Published:** 2016-09-22

**Authors:** Marjolein J Poen, Josanne H Verhagen, Ruth J Manvell, Ian Brown, Theo M Bestebroer, Stefan van der Vliet, Oanh Vuong, Rachel D Scheuer, Henk P van der Jeugd, Bart A Nolet, Erik Kleyheeg, Gerhard J D M Müskens, Frank A Majoor, Christian Grund, Ron A M Fouchier

**Affiliations:** 1 Erasmus MC, Department of Viroscience, Rotterdam, the Netherlands; 2 These authors contributed equally to this work; 3 Animal and Plant Health Agency (APHA) - Weybridge, Addlestone, Surrey, United Kingdom; 4 Netherlands Institute of Ecology (NIOO-KNAW), Department of Animal Ecology, Wageningen, the Netherlands; 5 Vogeltrekstation – Dutch Centre for Avian Migration and Demography (NIOO-KNAW), Wageningen, the Netherlands; 6 University of Amsterdam, Institute of Biodiversity and Ecosystem Dynamics, Computational Geo-Ecology, Amsterdam, the Netherlands; 7 Alterra, Center for Ecosystem Studies, Wageningen University, Wageningen, the Netherlands; 8 Sovon, Dutch Centre for Field Ornithology, Nijmegen, the Netherland; 9 Friedrich-Loeffler-Institut, Greifswald - Insel Riems, Germany

**Keywords:** avian influenza, emerging or re-emerging diseases, epidemiology, outbreaks, surveillance, viral infections

## Abstract

In 2014, H5N8 clade 2.3.4.4 highly pathogenic avian influenza (HPAI) viruses of the A/Goose/Guangdong/1/1996 lineage emerged in poultry and wild birds in Asia, Europe and North America. Here, wild birds were extensively investigated in the Netherlands for HPAI H5N8 virus (real-time polymerase chain reaction targeting the matrix and H5 gene) and antibody detection (haemagglutination inhibition and virus neutralisation assays) before, during and after the first virus detection in Europe in late 2014. Between 21 February 2015 and 31 January 2016, 7,337 bird samples were tested for the virus. One HPAI H5N8 virus-infected Eurasian wigeon (*Anas penelope*) sampled on 25 February 2015 was detected. Serological assays were performed on 1,443 samples, including 149 collected between 2007 and 2013, 945 between 14 November 2014 and 13 May 2015, and 349 between 1 September and 31 December 2015. Antibodies specific for HPAI H5 clade 2.3.4.4 were absent in wild bird sera obtained before 2014 and present in sera collected during and after the HPAI H5N8 emergence in Europe, with antibody incidence declining after the 2014/15 winter. Our results indicate that the HPAI H5N8 virus has not continued to circulate extensively in wild bird populations since the 2014/15 winter and that independent maintenance of the virus in these populations appears unlikely.

## Introduction

Wild birds are the natural hosts of low pathogenic avian influenza (LPAI) viruses, which generally do not cause clinical signs of disease in these host species [1]. So far, virus subtypes H1 to H16 and N1 to N9 have been detected in wild birds, of which viruses of subtypes H5 and H7 have shown the ability to evolve to highly pathogenic avian influenza (HPAI) viruses in poultry, causing severe disease with high mortality in such animals. These HPAI viruses were historically mainly detected in rapidly contained sporadic outbreaks in poultry, until H5N1 viruses of the A/Goose/Guangdong/1/1996 (GsGd) lineage emerged in Asia in 1997. Subsequently, these viruses have continuously circulated in poultry with frequent detections in wild birds [2] and with significant expansion in global range.

HPAI H5N8 viruses of the GsGd lineage of clade 2.3.4.4 emerged in poultry and wild birds on multiple continents in 2014. The ancestral influenza H5N8 virus to the strains causing outbreaks from 2014 onwards was first detected in China in 2010 in a captive-held mallard *(Anas platyrhynchos)* [3]. In early 2014, HPAI H5N8 GsGd virus of clade 2.3.4.4 occurred for the first time in poultry in South Korea, soon after causing outbreaks also in Japan [4]. From late 2014 onwards, this virus spread to other areas of the world including Europe, North America, Russia and Taiwan [5-8]. The HPAI H5N8 virus detections in Europe were limited to sporadic cases in wild birds and a relatively small number of unrelated outbreaks in poultry. However in North America HPAI H5N8 viruses reassorted with co-circulating LPAI viruses, giving rise to new HPAI H5N1 and H5N2 virus subtypes that caused a large number of outbreaks in poultry with numerous detections in wild birds [9]. Despite mild clinical symptoms caused by infection with HPAI H5N8 viruses of clade 2.3.4.4 in experimentally infected mammals [10-12] and ducks [11], the widespread detection and rapid global spread of HPAI H5 clade 2.3.4.4 viruses pose a potential threat to domestic and wild animals and should be studied further.

The major challenges in understanding the epidemiology of emerging influenza viruses in wild birds are the large numbers of potential host species and the usually short period of viral shedding, combined with the difficulty of catching and sampling representative numbers per species. For instance, mallards that were experimentally infected with HPAI H5N8 virus shed infectious virus in tracheal swabs for only up to 5 days post infection [11]. These impediments result in a low probability of detecting newly emerging avian influenza viruses in wild birds through active virological surveillance and result in a delay of implementation of effective control measures. Nevertheless, to date HPAI H5N8 virus has been detected in 30 wild bird species. In addition to the host species previously described [13,14], HPAI H5N8 viruses have been detected in wild bird species belonging to the orders *Anseriformes* in Asia (*Aythya* spp.) and North America (*Branta* spp.) [6]. In Europe, HPAI H5N8 viruses have been detected in bird species of the orders *Anseriformes* (*Anas* spp. and *Cygnus* spp.) and *Charadriiformes* (*Larus* spp.) [5,6,14].

To estimate the likelihood of the involvement of live wild birds in local and long distance movement of HPAI H5 viruses, information on recent exposure of wild bird populations to HPAI H5N8 viruses using serology, in addition to virology, would add substantial power to surveillance programmes. Studies with ferret sera have shown serological tests to have substantial discriminative power between antibodies directed to HPAI H5 viruses of different clades and LPAI H5 viruses using haemagglutination inhibition (HI) assays [12,15]. Although less is known about serology in wild birds, a study on wild birds sampled in Europe and Mongolia showed that antigenic differences between the haemagglutinin (HA) of classical Eurasian LPAI H5 viruses and GsGd lineage HPAI H5 viruses can be used to define bird populations in which HPAI viruses have previously been circulating [16]. With regard to HPAI H5N8 viruses specifically, a 2014 South Korean serology study showed evidence of a rise of H5 virus antibodies occurring in long distance migratory duck species after the onset of the HPAI H5N8 virus emergence in South Korea [4].

In this study, in response to the emergence of HPAI H5N8 virus in Europe, we present data on wild bird surveillance activities in the Netherlands, including results of virological and serological assays. 

## Methods

### Ethical statement

The capture of free-living birds was approved by the Dutch Ministry of Economic Affairs based on the Flora and Fauna Act (permit number FF/75A/2009/067 and FF/75A/2014/054). Handling and sampling of free-living birds was approved by the Animal Experiment Committee of the Erasmus Medical Centre (permit number 122–11–31). Free-living birds were released into the wild after sampling and all efforts were made to minimise animal suffering throughout the studies.

### Study population

Immediately after the first detection of HPAI H5N8 virus in poultry in Europe, ongoing influenza surveillance activities in migrating and overwintering wild birds in the Netherlands were intensified (14 November 2014–13 May 2015). Hereafter, this period will be referred to as ‘during the outbreak’. Surveillance activities in wild birds in the Netherlands were again intensified from the onset of the arrival of wild migrating birds a year after the initial HPAI H5N8 virus detection in Europe (1 September–31 December 2015). This period will be referred to as ‘after the outbreak’. Sampled populations consisted of resident birds, partial migrants and long distance migrants. During both periods of intensified surveillance, blood samples were obtained in addition to samples for virus detection. A matching historical set of serum samples was compiled based on similarity in species and family, hereafter referred to as ‘before the outbreak’ (2007–2013).

### Sample collection

Wild birds were captured using duck decoys, clap nets, cannon nets, mist nets, leg-nooses, swan hooks, or manually. Birds were sampled routinely for virus detection using cloacal and/or oropharyngeal swabs as described elsewhere [14]. In addition, faecal samples were collected from a limited number of species for virus detection. Blood samples were collected for antibody detection. Blood samples were collected from the brachial or metatarsal vein and centrifuged at 3,000 rpm for 10 min in 0.8 mL gel separation tubes (MiniCollect tubes, Roche). Serum samples were stored below -20 °C until analysis.

### Virus detection, isolation and characterisation

Samples for virus detection were analysed for the presence of HPAI H5(N8) virus using matrix- and H5-specific real-time polymerase chain reaction (RT-PCR) assays followed by H5 and neuraminidase sequencing as previously described [14]. Samples testing positive in matrix specific RT-PCR were inoculated in embryonated chicken eggs as described previously [17].

### Antibody detection

Serum samples were first tested for the presence of H5-specific antibodies in an HI assay according to standard procedures [18]. Briefly, serum samples were incubated for 16 hours at 37 °C with *Vibrio cholerae* filtrate containing receptor-destroying enzyme to remove non-specific inhibitors of haemagglutination activity, followed by incubation for 1 hour at 56 °C. Twofold serial dilutions of serum samples with a start dilution of 1:20 were prepared using phosphate-buffered saline (PBS) in U-bottomed 96 well microtitre plates. Serum dilutions were incubated with four haemagglutinating units (HAU) of Madin–Darby canine kidney (MDCK) (all HPAI H5 clade viruses) or egg (A/Mallard/Netherlands/3/1999) cultured virus for 30 min at 37 °C. A suspension of 1% turkey red blood cells (TRBC) was added to the serum-virus dilutions. After incubation for 1 hour at 4 °C, haemagglutination patterns were read. Negative controls, based on serum incubation without virus, were used to measure non-specific haemagglutination of each serum sample. Sera showing high background (i.e. high non-specific haemagglutination) were pre-treated with 10% TRBC for 1 hour at 4 °C and retested for the presence of H5-specific antibodies as described above. Serum samples from experimentally inoculated ferrets [12,15], a domestic duck, and a domestic goose were used as positive controls.

All serum samples were initially screened for antibodies specific for classical Eurasian LPAI H5N2 virus A/Mallard/Netherlands/3/1999 and clade 2.3.4.4 HPAI H5N8 virus A/Chicken/Netherlands/EMC-3/2014. Serum samples that tested positive for HPAI H5 clade 2.3.4.4-specific antibodies were further tested against HPAI viruses of the H5 clades 1 (A/Viet Nam/1194/2004), 2.1 (A/Indonesia/5/2005), 2.2 (A/Turkey/Turkey/1/2005), and 2.3 (A/Anhui/1/2005), and retested against the clade 2.3.4.4 virus. Samples showing more than threefold differences in titre or testing negative in the second assay after showing initial titres were tested a third time. The viruses used were recombinant viruses based on an A/PR/8/34 virus backbone, containing the HA and neuraminidase (NA) of the representative H5 strains. The sequences of the HA genes were modified to remove the multi-basic cleavage site to enable this study within biosafety level 2 laboratories. HPAI H5 virus of clade 0 was excluded from the analyses due to high overall reactivity with all avian positive control sera as previously described [16] and thus of limited discriminative value.

A representative selection (based on titre and serum availability) of serum samples that tested positive for HPAI H5 clade 2.3.4.4 antibodies were sent to the Animal and Plant Health Agency (APHA) (Weybridge, UK) for confirmation of HPAI H5 clade 2.3.4.4-specific antibodies using an HI assay. The HI assay procedure used by the APHA differed from the HI assay described above and was carried out in accordance to the World Organisation for Animal Health (OIE) [19]. In short, twofold serial dilutions of serum samples with a start dilution of 1:12 were made using phosphate-buffered saline (PBS) and prepared in V-bottomed microtitre plates. Serum dilutions were incubated with four HAU of egg cultured virus for 30 min at room temperature. A solution of 1% chicken red blood cells (CRBC) was added to the serum–virus dilutions. After incubation for 30 min at room temperature, haemagglutination patterns/streaming of red cells were read. Polyclonal chicken sera raised against the same clade 2.1, 2.2, 2.3, and 2.3.4.4 viruses as mentioned above were used as positive controls, supplemented with LPAI H5N3 virus A/Teal/England/7394–2805/2006 and clade 2.3.4.4 HPAI H5N8 virus A/Duck/England/36254/2014.

All samples that tested positive for HPAI H5 clade 2.3.4.4-specific antibodies in the initial HI assay were tested in a virus neutralisation (VN) assay if sufficient amounts of serum were available. The VN assay was performed as described previously [20], using titrated virus stocks of clade 2.1, 2.3, and 2.3.4.4. Briefly, serum was heat inactivated for 30 min at 56 °C and twofold serial dilutions of the sera starting at a 1:20 dilution were prepared and 100 median tissue culture infectious dose (TCID_50_) was added. After incubating antigen and serum for 1 hour at 37 °C with 5% CO_2_, the mixtures were transferred to 96 well flat bottom plates containing MDCK cells, which were washed once with infection medium before inoculation. The plates were incubated for 1 hour at 37 °C with 5% CO_2,_ after which the cells were washed once with 100 μL infection medium and the medium was replaced by 200 μL infection medium. Three days later, a haemagglutination assay was performed with the supernatant to determine the antibody titres.

## Results

### Study population

A total of 11,355 birds were sampled for virus detection during and after the first detection of HPAI H5N8 viruses in poultry and wild birds in Europe. Of those, 5,387 birds were sampled during the outbreak and 5,968 after the outbreak. This report describes the results on 7,337 samples obtained from 21 February 2015 onwards in addition to the previously reported 4,018 samples obtained until 20 February 2015 [14]. Sampled species mainly belonged to the orders *Anseriformes*, *Charadriiformes* and *Gruiformes* (Table 1). 

**Table 1:** Wild bird species sampled for virus detection during and after the emergence of highly pathogenic avian influenza H5N8 virus in Europe, the Netherlands, 21 February 2015–31 January 2016 (n = 7,337 animals)

Order	Family	Species	During outbreak: 21 Feb 2015–13 May 2015				After outbreak: 14 May 2015–31 Jan 2016			
			Birds sampled N	AIV-positive birds N	H5-positive birds N	Pathotype	Birds sampled N	AIV-positive birds N	H5-positive birds N	Pathotype
*Anseriformes*	Ducks	Common pochard *(Aythya ferina)*	0	0	0	NA	1	0	0	NA
		Common teal *(Anas crecca)*	8	0	0	NA	221	39	4	LPAI
		Egyptian goose *(Alopochen aegyptiaca)*	58	0	0	NA	136	0	0	NA
		Eurasian wigeon *(Anas penelope)*	175	1	1	HPAI	1,034	101	2	LPAI
		Gadwall *(Anas strepera)*	1	0	0	NA	175	15	0	NA
		Mallard *(Anas platyrhynchos)*	748	50	0	NA	2,464	354	15	LPAI
		Mandarin duck *(Aix galericulata)*	2	0	0	NA	0	0	0	NA
		Northern pintail *(Anas acuta)*	0	0	0	NA	7	3	0	NA
		Northern shoveler *(Anas clypeata)*	0	0	0	NA	17	2	0	NA
		Tufted duck *(Aythya fuligula)*	0	0	0	NA	1	0	0	NA
	Geese	Barnacle goose *(Branta leucopsis)*	96	5	4	LPAI	926	3	0	NA
		Bean goose *(Anser fabalis)*	0	0	0	NA	8	0	0	NA
		Brent goose *(Branta bernicla)*	54	0	0	NA	0	0	0	NA
		Canada goose *(Branta canadensis)*	3	0	0	NA	72	0	0	NA
		Greylag goose *(Anser anser)*	59	0	0	NA	239	0	0	NA
		Pink-footed goose *(Anser brachyrhynchus)*	0	0	0	NA	1	0	0	NA
		Greater white-fronted goose *(Anser albifrons)*	0	0	0	NA	55	0	0	NA
	Swans	Mute swan *(Cygnus olor)*	3	0	0	NA	31	1	0	NA
*Charadriiformes*	Gulls	Black-headed gull *(Chroicocephalus ridibundus)*	84	0	0	NA	392	53	0	NA
		Caspian gull *(Larus cachinnans)*	4	0	0	NA	4	0	0	NA
		Common gull *(Larus canus)*	1	0	0	NA	18	0	0	NA
		Great black-backed gull *(Larus marinus)*	1	0	0	NA	0	0	0	NA
		Herring gull *(Larus argentatus)*	15	0	0	NA	32	2	0	NA
		Lesser black-backed gull *(Larus fuscus)*	0	0	0	NA	33	2	0	NA
		Mediterranean gull *(Larus melanocephalus)*	1	0	0	NA	3	1	0	NA
		Yellow-legged gull *(Larus michahellis)*	0	0	0	NA	1	0	0	NA
	Lapwings	Northern lapwing *(Vanellus vanellus)*	6	0	0	NA	0	0	0	NA
	Terns	Black tern *(Chlidonias niger)*	0	0	0	NA	0	0	0	NA
		Common tern *(Sterna hirundo)*	0	0	0	NA	0	0	0	NA
*Columbiformes*	Pigeons	Common wood-pigeon *(Columba palumbus)*	1	0	0	NA	0	0	0	NA
*Gruiformes*	Coots	Common coot *(Fulica atra)*	46	0	0	NA	92	0	0	NA
	Rails	Little crake *(Porzana parva)*	0	0	0	NA	1	0	0	NA
		Common moorhen *(Gallinula chloropus)*	3	0	0	NA	4	0	0	NA
Total			1,369	56	5	NA	5,968	576	21	NA

AIV: avian influenza virus; HPAI: highly pathogenic avian influenza; LPAI: low pathogenic avian influenza; N: number; NA: not applicable.

Surveillance activities were intensified from 21 February to 13 May 2015 (n = 1,369) and 1 September to 31 December 2015 (n = 3,736).

For antibody detection, 1,443 serum samples were analysed. Among these, 945 samples from 25 avian species were obtained during the outbreak, while 349 samples from 15 species originated from after the outbreak. A total of 149 serum samples from 15 species sampled before the HPAI H5N8 virus emergence, obtained between 2007 and 2013, served as controls (Table 2). The majority of these samples were collected from birds wintering in Dutch wetlands.

**Table 2:** Wild bird species sampled for H5-specific antibody detection before, during and after the emergence of highly pathogenic avian influenza H5N8 virus in Europe, the Netherlands, 2007–2015 (n = 1,443)

Order	Family	Species	Number of individuals sampled		
			Before outbreak (before 2014)	During outbreak (14 Nov 2014–13 May 2015)	After outbreak (1 Sep 2015–31 Dec 2015)
*Anseriformes*	Ducks	Common teal *(Anas crecca)*	0	15	111
		Egyptian goose *(Alopochen aegyptiaca)*	9	62	28
		Eurasian wigeon *(Anas penelope)*	0	78	46
		Gadwall *(Anas strepera)*	1	3	1
		Mallard *(Anas platyrhynchos)*	21	93	18
		Mandarin duck *(Aix galericulata)*	1	2	0
		Northern pintail *(Anas acuta)*	0	0	1
		Northern shoveler *(Anas clypeata)*	0	2	3
		Ruddy shelduck *(Tadorna ferruginea)*	1	0	0
	Geese	Barnacle goose *(Branta leucopsis)*	20	19	0
		Bean goose *(Anser fabalis)*	5	0	0
		Brent goose *(Branta bernicla)*	0	19	0
		Greylag goose *(Anser anser)*	0	2	0
		Lesser white-fronted goose *(Anser erythropus)*	0	3	0
		Pink-footed goose *(Anser brachyrhynchus)*	0	1	0
		Greater white-fronted goose *(Anser albifrons)*	20	77	0
	Swans	Bewick's swan *(Cygnus columbianus bewickii)*	0	20	0
		Mute swan *(Cygnus olor)*	10	90	29
		Whooper swan *(Cygnus cygnus)*	0	1	0
*Charadriiformes*	Gulls	Black-headed gull *(Chroicocephalus ridibundus)*	20	262	31
		Caspian gull *(Larus cachinnans)*	0	6	3
		Common gull *(Larus canus)*	12	34	17
		Great black-backed gull *(Larus marinus)*	0	1	0
		Herring gull *(Larus argentatus)*	7	61	28
		Lesser black-backed gull *(Larus fuscus)*	1	3	8
		Mediterranean gull *(Ichthyaetus melanocephalus)*	2	1	0
		Yellow-legged gull *(Larus michahellis)*	0	0	1
*Gruiformes*	Rails	Common coot *(Fulica atra)*	19	84	24
		Moorhen *(Gallinula chloropus)*	0	6	0
Total			149	945	349

### Virus detection, isolation and characterisation

In addition to the two previously reported HPAI H5N8 virus-infected Eurasian wigeons detected in the Netherlands in November 2014 [14], the virus was detected in a third Eurasian wigeon faecal sample obtained on 25 February 2015 (1/1,369 birds sampled in 21 February–13 May 2015), near Ilpendam (52°28′N 4°57′E) (GenBank accession numbers: AKH14448–AKH14459). Since then, no HPAI H5N8 virus has been detected in any of the samples tested (0/5,968 birds sampled in 14 May 2015–31 January 2016) (Table 1).

### Influenza A H5 virus clade-specific antibody detection

As shown previously, ferret antisera raised against prototype strains representing LPAI and HPAI H5 viruses of various clades showed almost exclusive reactivity with homologous viruses in HI assays [12] (Table 3). Importantly, a ferret antiserum raised against the clade 2.3.4.4 virus did not react with other H5 viruses, and antisera raised against other prototype H5 strains did not react with the clade 2.3.4.4 virus A/Chicken/Netherlands/EMC-3/2014. Sera obtained upon inoculation of a domestic duck and a domestic goose with the clade 2.3.4.4 virus A/Turkey/Germany/AR2487/2014 reacted similar to the ferret clade 2.3.4.4 antiserum; no cross-reactivity was seen with other prototype H5 strains (Table 3). These data indicate that the antigenic differences between clade 2.3.4.4 HA and HA of LPAI and HPAI viruses belonging to other clades were sufficiently large to allow serological discrimination by HI assay.

**Table 3:** Details of positive control sera titres from experimentally infected ferrets, a domestic duck, and a domestic goose with one low pathogenic (LPAI) H5 and different highly pathogenic avian influenza (HPAI) H5 clades (n = 8 antisera)

Antiserum raised against	Characteristics	Species	Haemagglutination inhibition assay						Virus neutralisation assay		
			Viruses						Viruses		
			LPAI	HPAI clade					HPAI clade		
				1^a^	2.1^b^	2.2^c^	2.3^d^	2.3.4.4^e^	2.1^b^	2.3^d^	2.3.4.4^e^
A/Mallard/Netherlands/3/1999	LPAI H5N2	Ferret	**160**	< 10	< 10	< 10	< 10	< 10	ND	ND	ND
A/Viet Nam/1194/2004	HPAI H5N1 clade 1	Ferret	< 10	**80**	< 10	< 10	< 10	< 10	ND	ND	ND
A/Indonesia/5/2005	HPAI H5N1 clade 2.1	Ferret	< 10	< 10	**120**	< 10	60	< 10	**80**	< 10	< 10
A/Turkey/Turkey/1/2005	HPAI H5N1 clade 2.2	Ferret	< 10	< 10	< 10	**1,280**	60	< 10	ND	ND	ND
A/Anhui/1/2005	HPAI H5N1 clade 2.3	Ferret	< 10	< 10	< 10	20	**320**	< 10	< 10	**160**	< 10
A/Chicken/Netherlands/EMC-3/2014	HPAI H5N8 clade 2.3.4.4	Ferret	< 10	< 10	< 10	< 10	< 10	**160**	< 10	< 10	**40**
Turkey/Germany/AR2487/2014	HPAI H5N8 clade 2.3.4.4	Domestic duck	< 10	< 10	< 10	< 10	< 10	160	ND	ND	ND
Turkey/Germany/AR2487/2014	HPAI H5N8 clade 2.3.4.4	Domestic goose	< 10	< 10	< 10	< 10	< 10	80	ND	ND	ND

HPAI: highly pathogenic avian influenza; LPAI: low pathogenic avian influenza; ND: not determined.

Lowest serum dilution tested was 10. Titres indicating the reactivity of sera to viruses homologous to the viruses, which the sera were raised against are in bold.

^a^ A/Viet Nam/1194/2004.

^b^ A/Indonesia/5/2005.

^c^ A/Turkey/Turkey/1/2005.

^d^ A/Anhui/1/2005.

^e^ A/Chicken/Netherlands/EMC-3/2014.

### Influenza A virus H5-specific antibody detection in wild birds

#### Haemagglutination inhibition assays

Of the serum samples initially tested in the HI assay with LPAI H5N2 (A/Mallard/Netherlands/3/1999) and HPAI H5 clade 2.3.4.4 H5N8 (A/Chicken/Netherlands/EMC-3/2014) virus, LPAI H5-specific antibodies were detected in 31 of 1,443 serum samples and HPAI H5 clade 2.3.4.4-specific antibodies in 53 of 1,443 serum samples (Table 4). Among these, seven samples tested positive for both LPAI H5- and HPAI H5 clade 2.3.4.4-specific antibodies. The incidence of LPAI H5-specific antibodies was similar before, during and after the HPAI H5N8 virus emergence in Europe (Fisher exact test, p = 0.76 before vs during the outbreak; p = 0.39 during vs after the outbreak), while HPAI H5 clade 2.3.4.4-specific antibodies were detected exclusively in sera from five bird species, obtained during and after the HPAI H5N8 virus emergence in Europe (Table 4, Table 5). The incidence of HPAI H5 clade 2.3.4.4-specific antibodies a year after the outbreak (10/329 (20 samples with high background excluded), 3.0%) was lower than during the outbreak (43/940 (5 samples with high background excluded), 4.6%) (Fisher exact test, p = 0.27).

**Table 4:** Detected haemagglutination inhibition antibody titres to low pathogenic avian influenza H5 virus^a^ and to highly pathogenic avian influenza H5 clade 2.3.4.4 H5N8 virus^b^ in birds, before, during, and after detection of the highly pathogenic avian influenza H5N8 virus in Europe, the Netherlands, 2007–2015 (n = 1,443 birds)

Strain	Period relative to the outbreak^c^	Haemagglutination inhibition titre							High background	Total tested	Total positives
		BLD	10–40	40–80	80–160	160–320	320–640	≥ 640			
LPAI H5N2^a^	Before	121	1	0	1	0	0	0	26	149	2
	During	903	16	5	2	1	0	0	18	945	24
	After	324	2	1	0	2	0	0	20	349	5
HPAI H5N8^b^	Before	123	0	0	0	0	0	0	26	149	0
	During	897	7	20	6	4	5	1	5	945	43
	After	319	4	3	2	1	0	0	20	349	10

BLD: below limit of detection; LPAI: low pathogenic avian influenza; HPAI: highly pathogenic avian influenza.

Lowest serum dilution tested was 10.

^a^ A/Mallard/Netherlands/3/1999.

^b^ A/Chicken/Netherlands/EMC-3/2014.

^c^ The ‘outbreak’ refers to the six months following the detection of the highly pathogenic avian influenza H5N8 virus in Europe and this extends from 14 November 2014 to 13 May 2015. The period before the ‘outbreak’ is from 2007 to 2013, while the period after the ‘outbreak’ is from 1 September to 31 December 2015.

**Table 5:** Birds species with antibodies to highly pathogenic avian influenza H5 clade 2.3.4.4 H5N8 virus^a^, and number of respective animals, according to their haemagglutination inhibition antibody titres to the virus, during and after detection of highly pathogenic avian influenza H5N8 virus in Europe, the Netherlands, 14 November 2014–31 December 2015 (n = 382 birds)

Species	Period relative to the outbreak^b^	HI titre to HPAI H5 clade 2.3.4.4 (H5N8) virus							High background	Total tested
		BLD	10–40	40–80	80–160	160–320	320–640	≥ 640		
Eurasian wigeon (*Anas penelope*)	During	66	6	4	2	0	0	0	0	78
Lesser white-fronted goose (*Anser erythropus*)	During	2	0	1	0	0	0	0	0	3
Mute swan (*Cygnus olor*)	During	59	1	14	4	4	5	1	2	90
Common coot (*Fulica atra*)	During	83	0	1	0	0	0	0	0	84
Eurasian wigeon (*Anas penelope*)	After	42	2	1	0	0	0	0	1	46
Egyptian goose (*Alopochen aegyptiaca*)	After	27	1	0	0	0	0	0	0	28
Mute swan (*Cygnus olor*)	After	19	1	2	2	0	0	0	5	29
Common coot (*Fulica atra*)	After	21	0	0	0	1	0	0	2	24

BLD: below limit of detection; HI: haemagglutination inhibition; HPAI: highly pathogenic avian influenza.

Lowest serum dilution tested was 10.

^a^ A/Chicken/Netherlands/EMC-3/2014.

^b^ The ‘outbreak’ refers to the six months following the detection of the highly pathogenic avian influenza H5N8 virus in Europe and this extends from 14 November 2014 to 13 May 2015. The period after the ‘outbreak’ is from 1 September to 31 December 2015.

Serum samples obtained during (43/940 (5 samples with high background excluded), 4.6%) and after (10/329 (20 samples with high background excluded), 3.0%) the outbreak that tested positive for HPAI H5 clade 2.3.4.4-specific antibodies were subsequently tested in an HI assay against prototype viruses of clades 1, 2.1, 2.2, 2.3, and 2.3.4.4. Of the sera collected during the outbreak, 29/90 mute swans (*Cygnus olor*), 12/78 Eurasian wigeons, 1/3 lesser white-fronted geese (*Anser erythropus*) and 1/84 common coots (*Fulica atra*) tested positive for HPAI H5 clade 2.3.4.4**-**specific antibodies (Table 5). In these HPAI H5 clade 2.3.4.4-specific antibody positive sera, no cross-reactivity was observed in sera of Eurasian wigeons (12/12) and the lesser white-fronted goose (1/1). In contrast, the common coot (1/1) serum showed an additional titre to the clade 2.3 virus and sera of mute swans showed cross-reactivity to clade 2.3 (27/29), 2.1 (23/29), 1 (9/29) and 2.2 (4/29) viruses. In the majority of samples (22/29), titres to clade 2.1 and 2.3 exceeded those detected to clade 2.3.4.4 (Table 6).

**Table 6:** Titres of confirmatory haemagglutination inhibition and virus neutralisation assays for sera positive for highly pathogenic avian influenza H5 clade 2.3.4.4-specific antibodies in the initial screening, the Netherlands, 14 November 2014–31 December 2015 (n = 53 serum samples)

Period	Species^a^	Haemagglutination inhibition assay												Virus neutralisation assay		
		Initial							Confirmatory							
		LPAI H5	HPAI clade						LPAI H5	HPAI clade				HPAI clade		
			1		2.1	2.2	2.3	2.3.4.4^b^		2.1	2.2	2.3	2.3.4.4	2.1	2.3	2.3.4.4
During the outbreak:2014/15	Eurasian wigeon	< 10	< 10	< 10		< 10	< 10	50	ND	ND	ND	ND	ND	ND	ND	ND
	Eurasian wigeon	< 10	< 10	< 10		< 10	< 10	100	ND	ND	ND	ND	ND	ND	ND	ND
	Eurasian wigeon	20	< 10	< 10		< 10	< 10	15	ND	ND	ND	ND	ND	ND	ND	ND
	Eurasian wigeon	< 10	< 10	< 10		< 10	< 10	60	ND	ND	ND	ND	ND	ND	ND	80
	Eurasian wigeon	< 10	< 10	< 10		< 10	< 10	20	< 6	< 6	< 6	< 6	< 6	ND	ND	20
	Eurasian wigeon	< 10	< 10	< 10		< 10	< 10	40	< 6	< 6	< 6	< 6	< 6	ND	ND	20
	Eurasian wigeon	< 10	< 10	< 10		< 10	< 10	25	ND	ND	ND	ND	ND	ND	ND	40
	Eurasian wigeon	< 10	< 10	< 10		< 10	< 10	15	ND	ND	ND	ND	ND	ND	ND	20
	Eurasian wigeon	< 10	< 10	< 10		< 10	< 10	15	ND	ND	ND	ND	ND	ND	ND	10
	Eurasian wigeon	< 10	< 10	< 10		< 10	< 10	20	ND	ND	ND	ND	ND	ND	ND	20
	Eurasian wigeon	< 10	< 10	< 10		< 10	< 10	40	< 6	< 6	< 6	< 6	< 6	ND	ND	40
	Eurasian wigeon	< 10	< 10	< 10		< 10	< 10	120	ND	ND	ND	ND	ND	ND	ND	160
	Common coot	< 10	40	< 10		< 10	30	40	ND	ND	ND	ND	ND	ND	ND	< 10
	Lesser white-fronted goose	20	< 10	< 10		< 10	< 10	70	ND	ND	ND	ND	ND	< 10	< 10	< 10
	Mute swan	< 10	120	320		< 30	640	40	ND	ND	ND	ND	ND	< 10	< 10	< 10
	Mute swan	< 10	160	160		< 30	640	200	ND	ND	ND	ND	ND	< 10	< 10	< 10
	Mute swan	< 30	< 180	320		< 180	960	60	ND	ND	ND	ND	ND	ND	ND	ND
	Mute swan	< 120	< 120	120		< 120	320	240	ND	ND	ND	ND	ND	< 10	< 10	80
	Mute swan	< 30	30	160		40	640	480	ND	ND	ND	ND	ND	< 10	< 10	60
	Mute swan	< 60	< 60	< 40		< 40	60	480	ND	ND	ND	ND	ND	< 10	< 10	240
	Mute swan	< 60	< 40	240		< 30	640	70	ND	ND	ND	ND	ND	< 10	< 10	< 10
	Mute swan	< 120	< 60	160		< 60	640	960	12	< 6	< 6	< 6	192	< 10	10	240
	Mute swan	< 10	< 40	320		< 40	1,280	70	ND	ND	ND	ND	ND	< 10	< 10	< 10
	Mute swan	< 10	60	480		30	2,560	60	ND	ND	ND	ND	ND	< 10	< 10	< 10
	Mute swan	< 30	< 120	240		< 120	480	70	ND	ND	ND	ND	ND	ND	ND	< 10
	Mute swan	< 60	< 120	320		< 120	640	50	ND	ND	ND	ND	ND	< 10	< 10	< 10
	Mute swan	< 60	< 120	320		< 120	640	80	ND	ND	ND	ND	ND	< 10	< 10	20
	Mute swan	< 10	< 60	320		< 60	960	70	ND	ND	ND	ND	ND	< 10	< 10	< 10
	Mute swan	< 120	160	640		30	2,560	240	< 6	< 6	< 6	< 6	< 6	< 10	< 10	< 10
	Mute swan	< 60	40	320		30	1,280	120	ND	ND	ND	ND	ND	< 10	< 10	< 10
	Mute swan	< 30	30	160		< 30	640	50	ND	ND	ND	ND	ND	< 10	< 10	< 10
	Mute swan	< 10	ND	ND		ND	ND	50	ND	ND	ND	ND	ND	ND	ND	20
	Mute swan	< 120	< 120	160		< 120	640	70	ND	ND	ND	ND	ND	20	< 10	< 10
	Mute swan	< 10	160	320		< 120	1,280	70	ND	ND	ND	ND	ND	ND	ND	ND
	Mute swan	< 60	< 120	160		< 120	640	50	ND	ND	ND	ND	ND	< 10	< 10	< 10
	Mute swan	< 30	40	160		< 60	640	50	ND	ND	ND	ND	ND	< 10	< 10	< 10
	Mute swan	< 30	< 30	160		< 30	320	35	ND	ND	ND	ND	ND	< 10	< 10	< 10
	Mute swan	< 10	< 180	320		< 180	640	100	ND	ND	ND	ND	ND	< 10	< 10	< 10
	Mute swan	40	< 60	160		< 60	640	80	ND	ND	ND	ND	ND	ND	ND	ND
	Mute swan	< 60	< 60	< 60		< 60	160	240	ND	ND	ND	ND	ND	< 10	< 10	10
	Mute swan	< 60	< 240	< 240		< 240	< 240	480	12	< 6	< 6	< 6	96	< 10	< 10	60
	Mute swan	< 60	< 30	< 30		< 30	60	480	< 6	< 6	< 6	< 6	96	< 10	< 10	240
	Mute swan	< 120	< 120	< 120		< 120	320	480	ND	ND	ND	ND	ND	< 10	< 10	60
After the outbreak: 2015	Eurasian wigeon	< 10	< 10	< 10		< 10	< 10	20	ND	ND	ND	ND	ND	ND	ND	160
	Eurasian wigeon	< 10	< 10	< 10		< 10	< 10	10	ND	ND	ND	ND	ND	ND	ND	20
	Eurasian wigeon	< 10	< 10	< 10		< 10	< 10	40	ND	ND	ND	ND	ND	ND	ND	80
	Common coot	< 10	80	60		60	320	160	ND	ND	ND	ND	ND	ND	ND	20
	Egyptian goose	< 10	< 10	< 10		< 10	80	25	ND	ND	ND	ND	ND	ND	ND	< 10
	Mute swan	160	80	60		< 30	160	120	ND	ND	ND	ND	ND	< 10	< 10	40
	Mute swan	40	80	80		80	320	45	ND	ND	ND	ND	ND	< 10	< 10	< 10
	Mute swan	< 10	< 10	< 10		< 10	30	15	ND	ND	ND	ND	ND	< 10	< 10	< 10
	Mute swan	< 10	80	80		80	320	60	ND	ND	ND	ND	ND	< 10	< 10	< 10
	Mute swan	< 10	160	160		240	320	80	ND	ND	ND	ND	ND	80	20	< 10

HPAI: highly pathogenic avian influenza; LPAI: low pathogenic avian influenza; ND: not determined.

Lowest serum dilution tested was 10 for the initial haemagglutination inhibition (HI) and virus neutralisation assay and 6 for the confirmatory HI assay.

^a^ Species included common coot (*Fulica atra*), Egyptian goose (*Alopochen aegyptiaca*), Eurasian wigeon (*Anas penelope*), lesser white-fronted goose (*Anser erythropus*), mute swan (*Cygnus olor*).

^b^ Mean titre of in duplo tested samples.

Of the sera collected after the outbreak, 5/29 mute swans, 3/46 Eurasian wigeons, 1/28 Egyptian geese (*Alopochen aegyptiaca*) and 1/24 common coots tested positive for HPAI H5 clade 2.3.4.4**-**specific antibodies (Table 5). The sera of the Eurasian wigeons reacted with HPAI H5N8 virus exclusively. However, the common coot as well as 1/5 mute swans showed HI titres to all five H5 clades. The other 3/5 mute swans showed HI titres to multiple but not all H5 clades, while 1/5 mute swans and 1/1 Egyptian goose only showed an additional titre to clade 2.3 (Table 6).

Seven of the HPAI H5 clade 2.3.4.4-seropositive bird sera obtained during the outbreak, from four mute swans and three Eurasian wigeons, were retested in an HI assay at the APHA. Here, 3/4 mute swan samples with high initial HI antibody titres against HPAI H5 clade 2.3.4.4 (H5N8) virus were confirmed. However, 1/4 mute swan sera could not be confirmed, and HPAI H5 clade 2.3.4.4-specific antibodies were also not detected in 3/3 sera of the Eurasian wigeons that had low antibody titres in the initial tests (Table 6).

#### Virus neutralisation assays

For 37/43 HPAI H5 clade 2.3.4.4-positive sera collected during and 10/10 sera collected after the outbreak, sufficient serum volumes were available for retesting in a VN assay. In this assay, HPAI H5 clade 2.3.4.4-specific antibodies were detected in sera of 9/9 Eurasian wigeons and of 10/26 mute swans obtained during the outbreak. Sera of the mute swans did not react with viruses of other H5 clades. HPAI H5 clade 2.3.4.4-specific antibodies were not detected in the sera of the common coot and the lesser white-fronted goose by VN assay. HPAI H5 clade 2.3.4.4-specific antibodies were confirmed by VN assay in sera from 3/3 Eurasian wigeons, 1/5 mute swans, 1/1 common coot and 0/1 Egyptian goose collected after the outbreak (Table 6).

## Discussion

In this report surveillance data for HPAI H5N8 in birds in the Netherlands are presented. In addition to bird samples previously investigated for the virus from 14 November 2014 to 20 February 2015, a new set of 7,337 samples obtained between 21 February 2015 and 31 January 2016 is analysed. One faecal sample obtained from a Eurasian wigeon (*Anas penelope*) on 25 February 2015 tested positive for the HPAI H5N8 virus, adding to the previous finding of the virus in two Eurasian wigeons in the country in late 2014 [14]. Virological surveillance moreover suggests that only very limited numbers of wild bird species were identified as potential hosts in Europe. Importantly, to the best of our knowledge, there are no reports of additional findings of HPAI H5N8 viruses in wild birds and poultry in Europe, since the last detection of the virus in February 2015 in the Netherlands. 

Given the difficulty of detecting newly emerging HPAI virus strains in wild birds however, the application of a more sensitive and cost-effective method to detect potential host species is warranted. For this purpose, we performed serological assays specifically aimed to detect antibodies specific to HPAI H5 clade 2.3.4.4 viruses in a substantial number of sera obtained before, during, and after HPAI H5N8 emergence in the Netherlands. Three potential HPAI H5N8 host species were identified by HI assays and confirmed by VN assays; Eurasian wigeons, mute swans and common coots. Considering the results of virological studies performed worldwide since the onset of the HPAI H5N8 virus emergence in early 2014, the detection of HPAI H5 clade 2.3.4.4-specific antibodies in these species is not surprising. HPAI H5N8 virus was isolated from Eurasian wigeons in Russia [8] and the Netherlands [14], from mute swans in Sweden [6], and from a common coot in South Korea [21]. 

The serological results reported here were not entirely consistent between HI and VN assays and between HI assays performed in two different laboratories. Although low HI titres (e.g. in Eurasian wigeons) were reproducible within a laboratory with the same HI assay and a VN assay, they were not detected by HI assay in a second laboratory, potentially due to differences in the methods used and hence differences in sensitivity and specificity. High antibody titres in mute swan sera were reproduced by HI assay in a second laboratory and by VN assay, but low antibody titres in mute swans were not always reproduced. While it is thus clear that individual HI titres in avian sera obtained from a single test cannot be used reliably for diagnosis, use of serum panels from cohorts of birds, use of multiple tests to cross-validate results, a panel of relevant viruses and use of collections of control antisera may still enable the use of serological tests in support of HPAI H5 surveillance studies.

Previously, HI assays were shown to be discriminative enough to detect antibodies in serum samples collected from free-living wild birds in Europe and Asia to be directed to either HPAI or LPAI H5 viruses. However, widely varying results were obtained as far as HPAI H5 clade-specific antibodies were concerned [16]. In this study, most birds that tested positive for HPAI H5 clade 2.3.4.4-specific serum antibodies showed relative low HI titres. This is in accordance with findings based on experimental HPAI H5N8 virus infections of ferrets [10-12], possibly indicating low immunogenicity upon infection. In addition, there is limited knowledge about the longevity of avian antibodies after naturally occurring infection with avian influenza viruses. Antibodies specific to LPAI viruses were detected up to several months after experimental or natural infection [22-24], whereas little is known about the duration of detection of antibodies specific to HPAI viruses with a reported maximum of detection of 28 days after experimental infection in domestic ducks [25]. To date, there is no knowledge on the effect of a prior exposure to an unrelated subtype or on the phenomena of antigenic sin in avian species. Hypothetically, low immunogenicity in combination with decreasing titres in time could be an explanation for the low incidence and relative low titres of antibodies detected in wild bird sera in this study.

In conclusion, our results provide evidence that clinically unaffected long distance migratory and local wild birds sampled in the Netherlands during the H5N8 outbreak late 2014 and early 2015, and again late 2015, have been exposed to HPAI H5N8 or closely related HPAI H5 clade 2.3.4.4 viruses and seroconverted upon exposure. Since HPAI H5N8 virus has not been detected in Europe since early 2015 and because HPAI H5 clade 2.3.4.4-specific antibody incidence decreased in time, we conclude that the virus has not circulated extensively at the breeding grounds in summer and upon the return of the birds to their wintering areas in the 2015/16 winter. As a consequence, the newly emerging HPAI H5N8 clade 2.3.4.4 virus subtype appears to have already disappeared from European wild birds indicating that sustained transmission and independent maintenance may be less likely. This is an important consideration in the ongoing evolution and ecology of these viruses in wild birds and the potential risks they pose for introduction to poultry and the pathways through which they might spread. Finally we recommend that serological tools be further optimised, harmonised, and validated for avian influenza surveillance studies in wild birds.
